# Cold winters have morph-specific effects on natal dispersal distance in a wild raptor

**DOI:** 10.1093/beheco/arab149

**Published:** 2021-12-30

**Authors:** Arianna Passarotto, Chiara Morosinotto, Jon E Brommer, Esa Aaltonen, Kari Ahola, Teuvo Karstinen, Patrik Karell

**Affiliations:** University of Seville, Department of Zoology, Sevilla, Spain; Bioeconomy Research Team, Novia University of Applied Sciences, Raseborgsvägen 9, FI-10600 Raseborg, Finland; Bioeconomy Research Team, Novia University of Applied Sciences, Raseborgsvägen 9, FI-10600 Raseborg, Finland; Evolutionary Ecology Unit, Department of Biology, Lund University, Sölvegatan 39 (Ecology Building), SE-223 62 Lund, Sweden; Department of Biology, University of Turku, 20014 Turku, Finland; Vanhansahantie 13B 7, FI-08800 Lohja, Finland; Tornihaukantie 8D 72, FI-02620 Espoo, Finland; Juusinkuja 1, FI-02700 Kauniainen, Finland; Bioeconomy Research Team, Novia University of Applied Sciences, Raseborgsvägen 9, FI-10600 Raseborg, Finland; Evolutionary Ecology Unit, Department of Biology, Lund University, Sölvegatan 39 (Ecology Building), SE-223 62 Lund, Sweden

**Keywords:** climate change, distribution pattern, genotype by environment, movement ecology, melanism, population dynamics

## Abstract

Dispersal is a key process with crucial implications in spatial distribution, density, and genetic structure of species’ populations. Dispersal strategies can vary according to both individual and environmental features, but putative phenotype-by-environment interactions have rarely been accounted for. Melanin-based color polymorphism is a phenotypic trait associated with specific behavioral and physiological profiles and is, therefore, a good candidate trait to study dispersal tactics in different environments. Here, using a 40 years dataset of a population of color polymorphic tawny owls (*Strix aluco*), we investigated natal dispersal distance of recruiting gray and pheomelanic reddish-brown (hereafter brown) color morphs in relation to post-fledging winter temperature and individual characteristics. Because morphs are differently sensitive to cold winters, we predicted that morphs’ natal dispersal distances vary according to winter conditions. Winter temperature did not affect the proportion of brown (or gray) among recruits. We found that dispersal distances correlate with winter temperature in an opposite manner in the two morphs. Although the gray morph undertakes larger movements in harsher conditions, likely because it copes better with winter severity, the brown morph disperses shorter distances when winters are harsher. We discuss this morph-specific natal dispersal pattern in the context of competition for territories between morphs and in terms of costs and benefits of these alternative strategies. Our results stress the importance of considering the interaction between phenotype and environment to fully disentangle dispersal movement patterns and provide further evidence that climate affects the behavior and local distribution of this species.

## Teaser

In species displaying alternative colorations (morphs), climate may induce different responses according to the intensity of pigmentation. We show that brown, more pigmented, tawny owls tend to move away farthest from their place of birth in milder winters, whereas paler gray tawny owls move farthest in colder winters. The ongoing changes in winter severity will likely affect differently movement patterns of these two tawny owl color morphs, with important consequences on morph distribution and population dynamics.

## INTRODUCTION

Dispersal is a key process of population and evolutionary dynamics as it has major consequences on individual fitness and species distribution patterns (Bonte et al. 2012). To avoid intra- and interspecific competition for resources and/or mates, animals often have to disperse over long distances, which will influence individual reproductive output and survival probability (Ronce 2007, Clobert et al. 2009). Dispersal also affects the composition of the population by redistributing individuals not only spatially, but also temporally because it affects the number of individuals present in the population at a certain time (Benton and Bowler 2012). Dispersal also shapes the genetic structure of populations through gene flow due to emigration and immigration, which prevents ultimately the risk of inbreeding and extinction (Ronce 2007). Two main dispersal movements are recognized: breeding dispersal, indicating the movement of adults from different breeding sites between successive years, and natal dispersal, that is the displacement of pre-reproductive individuals from their natal home range to the territory where the first breeding event takes place (Greenwood and Harvey 1982). Natal dispersal accounts for the broadest movements, because distances traveled in breeding dispersal are usually shorter or null due to extensive reuse of territories (Greenwood and Harvey 1982), although opposite patterns are documented (Dale et al. 2005). Therefore, natal dispersal plays a more critical role in individual survivorship and population dynamics because longer distances entail higher mortality risk, potentially leading to lower fecundity (Waser et al. 2013), and loss of advantages derived by being locally adapted (Bonte et al. 2012).

Theory predicts that individual variation in natal dispersal is not merely the result of random decisions or inheritance, though evidence for heritability of natal dispersal variance has accumulated over the years (e.g. Hansson et al. 2003, Pasinelli et al. 2004, Matthysen et al. 2005, Charmantier et al. 2011, Saastamoinen et al. 2018). Rather, departure, vagrant stage, and settlement, which are the main phases of the dispersal process, would be dependent on multiple aspects of the environment (i.e. context-dependence) that, in turn, affect dispersers’ condition and their propensity to disperse (i.e. phenotype-dependence) (Clobert et al. 2009). In fact, natal dispersal within (Garant et al. 2005) and/or between populations (Senar et al. 2002, Tarwater and Beissinger 2012), can be simultaneously affected by individual (e.g. sex, body mass, and/or personality; Cote et al. 2010, Saino et al. 2014, Trochet et al. 2016), ecological (e.g. habitat quality and/or food abundance; Pasinelli and Walters 2002, Coles et al. 2003) and social (e.g. conspecifics density, Serrano et al. 2003) features. Assessing the relative importance of individual attributes versus environmental context is critical to get insight into the relative role of intrinsic and extrinsic factors in population dynamics (Rémy et al. 2011, Aguillon and Duckworth 2015, Fattebert et al. 2019, Baines et al. 2019). However, few studies have examined how phenotype and environmental conditions interact to influence natal dispersal (e.g. Benard and McCauley 2008, Tarwater and Beissinger 2012) even though the coexistence of different phenotypic traits can be closely linked with the evolution of different natal dispersal strategies when associated with additive genetic variance (Garant et al. 2005).

Melanin-based coloration is a highly heritable phenotypic trait that varies greatly within species among vertebrates (Mundy 2005, Hoekstra et al. 2006, Ducrest et al. 2008, McNamara et al. 2021). Given the detectable genetic basis of dispersal in some systems (Saastamoinen 2008), melanin-based coloration can be used to explore genetically based variation in natal dispersal. A shared pathway of melanin synthesis with the melanocortin system, which pleiotropically influences physiological and behavioral traits (Ducrest et al. 2008), has been suggested to explain why different degrees of melanization predict various personality attributes such as aggressiveness, boldness, and exploratory behavior (van den Brink et al. 2012b, Mateos-Gonzalez and Senar 2012, Nicolaus et al. 2016). These, in turn, can impact an individual’s dispersal strategy. Darker (i.e. more melanistic) individuals are generally bolder and show higher dispersal propensity (van den Brink et al. 2012a, Roulin 2013, Saino et al. 2014, Camacho et al. 2018). In addition to personality and dispersal propensity, melanin-based color polymorphism is also associated with the ability of different morphs to adapt to heterogeneous local environments (Dreiss et al. 2012, Passarotto et al. 2018). Indeed, because melanism has been coupled with the ability to cope with stressful processes, such as thermoregulation (Clusella-Trullas et al. 2008, Dreiss et al. 2016), we may also expect different responses in terms of movement in relation to climatic conditions (Roulin 2014). Accordingly, melanin-based color polymorphism could determine morph-specific dispersal patterns in relation to climate variation, a possibility so far overlooked.

Here, we aim to investigate the potential link between color polymorphism and natal dispersal variation in Tawny Owl (*Strix aluco*), a forest-dwelling, sedentary and nocturnal bird of prey widespread in Eurasia. This species displays a pheomelanin-based color polymorphism ranging from pale gray (hereafter “gray”) to a darker, more pheomelanic, reddish-brown (hereafter “brown”) morph, inherited according to a Mendelian one-locus-two allele pattern with brown dominance (Karell et al. 2011b, Morosinotto et al. 2020). Importantly, the morphs display different abilities to cope with environmental fluctuations, which are linked with different life-history strategies (Emaresi et al. 2011). The gray morph has a higher adult survival than the brown morph in cold and snow-rich winters (Karell et al. 2011b), which may be due to the gray morph’s better plumage insulation capacity (Koskenpato et al. 2016) and/or lower detection probability in snowy landscapes (Koskenpato et al. 2020). On the other hand, the brown morph consistently produce heavier offspring, which have a higher probability to recruit to the local population (Morosinotto et al. 2020) and brood size manipulation experiments suggest that brown parents have a higher fixed parental effort whereas gray parents have a more flexible parental effort depending on the conditions (Emaresi et al. 2014). During nestling growth, food requirements are morph-specific as gray offspring grow relatively better and mount higher immune responses under adverse food conditions and brown ones grow better and mount higher immune responses under favorable food conditions (Piault et al. 2009). The color morphs also reflect distinct physiological profiles in adulthood because brown tawny owls molt more primaries than gray ones (Karell et al. 2013), show higher costs of immune defense (Gasparini et al. 2009, Karell et al. 2011a) and a faster rate of cellular senescence than gray ones (Karell et al. 2017, Morosinotto et al. 2021).

We use a 40 years dataset to study local natal dispersal distances traveled by tawny owl color morphs in a study population close to the species’ colder range margin in relation to winter severity. We estimate local dispersal distance based on the distance between marking and recapture within our 500 km^2^ study area. We expect dispersal distances not to be strongly affected by the size of our study area because the tawny owl is highly sedentary and philopatric (Saurola et al. 2013), and therefore the majority of natal dispersal distances are expected to be within our study area. In addition, our main interest is to contrast the difference in dispersal between the morphs and we expect differences in mean natal dispersal distances to be apparent within our study area. In general, natal dispersal distance is expected to increase with winter severity if the owls are aiming to disperse to more favorable areas. Alternatively, owls born before a cold winter may disperse shorter distances because adult mortality is higher in harsh winter conditions (Karell et al. 2011b) and this is expected to create vacant territory opportunities. The brown morph has markedly lower survival than the gray in cold and snowy years as it is less tolerant to adverse, stressful, and cold environments (Karell et al. 2011b). We, therefore, predict that winter conditions would also have a different impact on morphs’ dispersal distance, due to the different abilities of the two morphs to cope with costs of dispersal under harsh winters conditions. Food availability may be another factor underpinning variation in natal dispersal (Coles et al. 2003). Age at first breeding in tawny owls depends on small mammal abundance (Karell et al. 2009, Millon et al. 2010) and may therefore also affect the distance they travel before settling. To assess this possibility, we also take into account small mammal prey abundance, predicting that adverse food resources would lead to increased natal dispersal distances. Finally, we also hypothesize that differences in fledglings’ body conditions may potentially result in natal dispersal distance variation (Fattebert et al. 2019), where heftier individuals would be more prone to disperse longer distances, whereas individuals in poorer conditions would avoid traveling long distances and settle closer to their natal territory.

## MATERIAL AND METHODS

### Study area

We studied natal dispersal in tawny owls in two almost overlapping and equally sized areas in western Uusimaa, Southern Finland (60°15′N, 24°15′E). The two localities extend over a total area of approximately 500 km^2^ and are equipped with about 360 nest-boxes (ca. 150 each, for description of the study areas, see Brommer et al. 2015, Morosinotto et al. 2020. See also Supplementary Figure S1). One population was monitored from 1978 to 2018 by authors KA, TK, and PK and the other between 1987 and 2018 by author EA. All nest boxes were associated with geographical coordinates, enabling us to compute linear distances between them.

### Study species

Tawny owls breed early in spring, generally laying two to six eggs, which are incubated by the female alone during roughly 30 days. Tawny owls display strong pair bonding between the social mates (Southern 1970, Hirons 1985, Jedrzejewski et al. 1996, Sunde 2011) and have biparental care with clear division of duties, where males are the main food provider for females and offspring, although females brood the chicks and allocate the food among them and defend the nest (Sunde et al. 2003). The young fledge in late spring/early summer when they are not fully able to fly and they stay in their natal territory with their parents for about three months. In early fall, they start dispersing (Mikkola 1983, König and Weick 2008, Sunde 2008). This period is critical with high mortality (e.g. Overskaug et al. 1999) and nothing is known about how long the vagrant and settling phases last. During natal dispersal, tawny owl juveniles tend to prefer areas of high vole abundance (Coles et al. 2003) though survival of adults is not dependent on vole abundance (Karell et al. 2009).

In Finland, tawny owls inhabit mixed and boreal forests where they readily breed in nest boxes. After a first preliminary check in early April, to achieve information on breeding attempts, nest boxes were regularly checked to record laying date, brood size, and biometrical measures. Both parents were trapped at the nest box during the nestling period, aged on the basis of plumage characteristics (tawny owls typically molt only part of their flight feathers; see Karell et al. 2013), measured (wing length and mass) and ringed to allow individual identification (Karell et al. 2009). Plumage color was scored in adults using a semi-continuous score based on the degree of pheomelanin-based redness from: facial disc, back, breast, and general appearance. Then, each individual was categorized as either gray or brown morph based on this overall score (see details in Brommer et al. 2005). All the offspring were ringed, weighed, and measured shortly before fledging, at roughly 25–28 days age. Local recruitment as breeding individuals was recorded when adult owls ringed as nestlings were caught breeding within the study area in following breeding seasons, because no capturing was performed outside the breeding season.

Overall, we recorded 192 recruited offspring out of the 3010 ringed offspring (6.38%, 1979–2018). The recruited individuals originated from 150 nests (100 females and 92 males; 134 gray and 56 brown, two not color scored). In this data set, we consider 89 individuals caught as one-year-old first breeders (46.4%), 59 two-year-old first breeders (30.7%), and 44 three-year-old or older first breeders (22.9%). Dispersal distances were considered not only according to body mass at fledging and morph, but also according to the winter temperature experienced during the first year post-fledging (i.e. early life conditions). To do that, for each study year we considered data on annual winter mean temperature (1. December–28. February), which was collected from the Finnish Meteorological Institute (FMI), from Helsinki-Vantaa airport weather station, situated circa 50 km east of the study area. We used the index “winter temperature anomaly” (i.e. where winters colder than average are assigned negative values), which is a widely used proxy calculated as the deviation of the average winter temperature from the long-term average in years 1981–2010.

Small mammal density was estimated each year in autumn, from 1981 onward, by snap trapping in two sites within the study area, each containing both an open (field/clear-cut) and a forest habitat. Traps were set as transects of 16 trapping spots with three traps each in both sites, and all triggered for two consecutive nights (192 traps each night = 384 trap nights in total). Prey abundance was calculated as the number of captures per 100 trap nights (see details in Karell et al. 2009).

Natal dispersal was calculated as the linear distance in km from the nest site of rearing to the breeding nest site. We assumed that dispersal could occur in any direction, because other studies found no clear directionality in dispersal in other species, other than those determined by geographical barriers and/ or environmental features (Mátics 2003, Delgado et al. 2010).

### Statistical analyses

Natal dispersal distances were modelled as Linear Mixed Models (LMMs), using the function “lmer” implemented in package “lme4” (Bates et al. 2007) in R (R Development team 2019), because residuals fitted normality assumption. Year and “brood ID”, which is a unique numerical code assigned to each brood in a given year to control for the statistical non-independence of sibling recruits, were included as random effects. Moreover, to account for variation in dispersal associated with the location of nest boxes in the study area, we included nest box identity (“Nest box ID”) as a random factor. To assess a predicted morph-specific dispersal strategy in relation to winter conditions, we considered morph (scored as a binary variable where 1 = gray, 2 = brown), winter temperature and their interaction as fixed factors. Furthermore, to control for the potential influence of food availability on dispersal behavior, we included small mammal density index. We also controlled for the impact of sex and fledgling body mass on natal dispersal distances because they could affect recruitment probability and distances. The factor “sex” was coded as 0 = female and 1 = male, whereas body mass was standardized to zero mean and unit standard deviation before analyses (Schielzeth 2010). Overall we considered in the analysis 180 individuals (out of 192; ca. 94% of recruits) for which we had all of this information. The degrees of freedom were calculated with Satterthwaite’s method.

Because age at first breeding (recruitment) varies depending on food conditions (Karell et al. 2009, Millon et al. 2010, see also Material and methods), we ran two different sets of models. First, we considered the larger data set (*n* = 180; see Table 1) where we checked if age at which they recruited had an effect on dispersal distance. In a second set, we consider only those individuals which recruited as one-year-olds (*n* = 84; see Table 1) to fully confirm any observed pattern (see Results).

**Table 1:** Table showing the breakdown of the sample sizes considered in each analysis

Data set (*n*)	Gray	Brown	Unknown	Analysis
192	134	56	2	Descriptive statistics
				GLM testing proportion of brown individuals in relation to winter temperature
				Randomization test
180	128	52	0	LMM (all ages, complete information)
84	65	19	0	LMM (one-year-old individuals, complete information)

Previous analyses showed that recruitment is not directly morph-dependent (Morosinotto et al. 2020). However, before main analyses, we performed a Generalized Linear Model (GLM) with binomial errors to test whether the proportion of individuals among the recruits in the whole sample (*n* = 190; see Table 1) was morph-specific according to average winter temperature experienced in their first winter. To this end, we used the proportion of brown individuals calculated for each year of birth as the dependent variable and post-fledging winter temperature anomaly as the explanatory variable.

### Estimation of possible detectable distances

Observational limits posed by the finite extent of the study area may bias the estimation of the dispersal distances because individuals traveling out of the study area are missed. Furthermore, the observed distribution of dispersal distances may also be influenced by other extrinsic factors, such as landscape configuration (Brommer and Fred 2007). To assess whether dispersal distances were biased by the spatial scale and our network of nest boxes, we made a comparison between expected and observed dispersal distances following the approach outlined in Brommer and Fred (2007). First, we estimated the possible detectable dispersal distances by computing pairwise distances between all the boxes present in the study area in these 40 years (*n* = 368) also separating boxes before and after 1987, when the study area was enlarged (see Study area description). Then, for each recruit, we computed the possible distances to disperse from the nest box of origin to all nest boxes. If a recruit hatched in 1987 or later we considered all boxes, otherwise only the boxes in the pre-1987 set were included. These analyses allowed us to assess the study area restrictions relying on a bootstrap approach. Through a randomization test based on 999 repetitions, we drew a random distance from all possible distances that was then compared with the expected median dispersal distance (computed from the 999 random draws). Under the assumption that the settlement of the owls is completely random in the study area, we predicted that the observed distance would fall within the distribution of expected dispersal distances under random draws. Hence, a randomized *P* value is the number of random median dispersal distances that is larger than the observed median distance divided by 1000 (999 random + 1 observed).

## RESULTS

### Variation in dispersal distance

**Figure 1:**
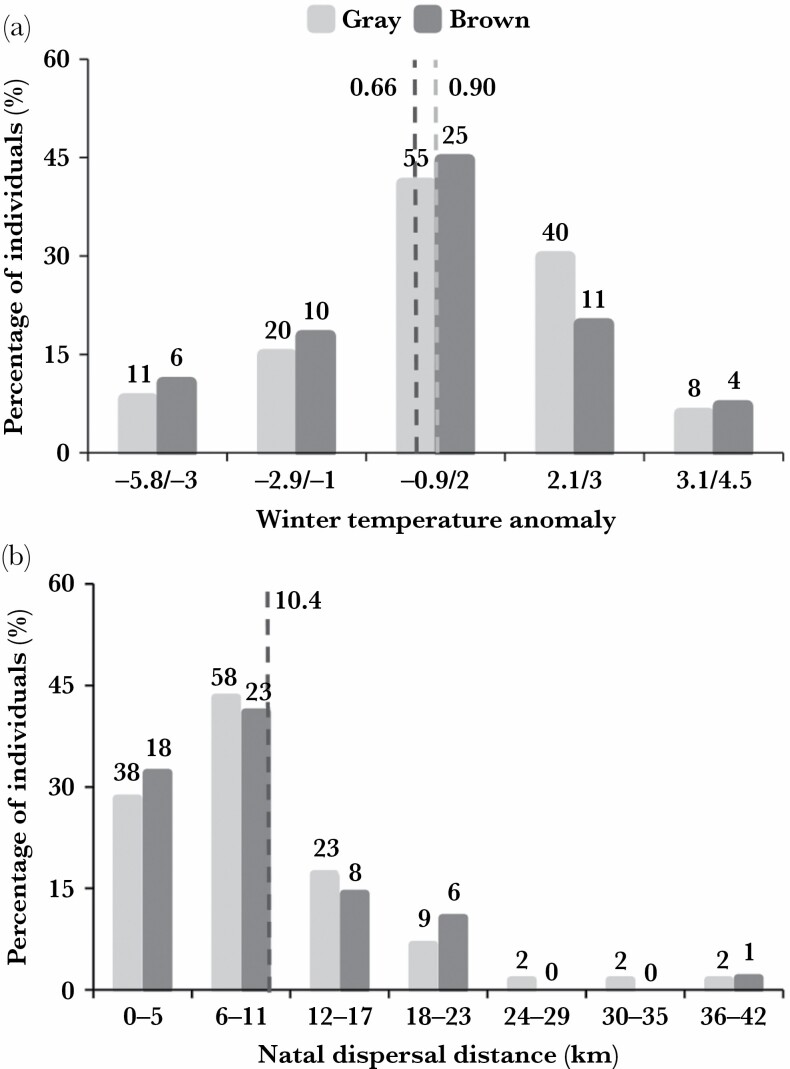
Number of recruits (%) according to morph in relation to (a) winter temperature anomaly and (b) dispersal distances (km). The dashed lines indicate average value of natal dispersal distances and winter temperature anomaly respectively for each morph (relative values are shown next to the lines; in panel (b) only one line is visible as both morphs traveled similar distances; see Results) (*n* = 190). Number of individuals are shown above bars.

**Figure 2:**
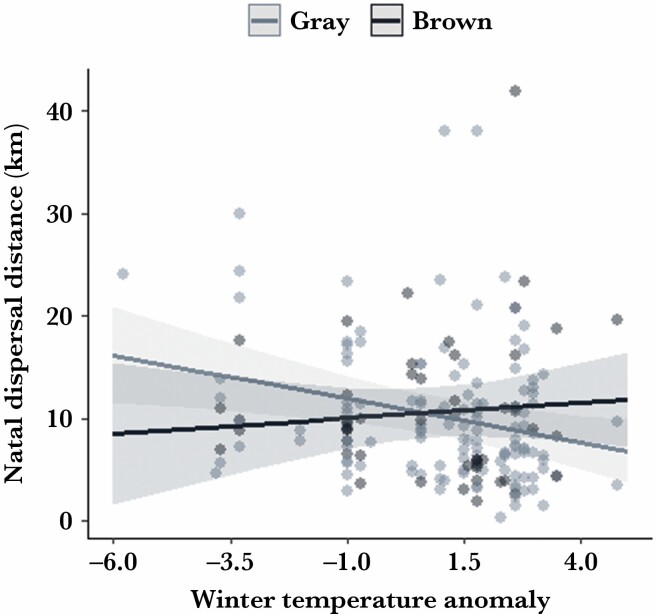
Dispersal distances traveled by gray and brown recruited individuals in relation to winter temperature anomaly (see Methods). Lighter gray dots indicate gray individuals (*n* = 128) whereas darker gray dots indicate brown individuals (*n* = 52). The plot presents the marginal effects (±95 CI) of the interaction in the statistical model (see Table 2a).

The yearly proportion of brown recruits did not depend on the winter temperature (binomial GLM: estimate ± SE = −0.07 ± 0.08, df = 1, 33, *z* value = −0.88, *P* = 0.38; Figure 1a). Overall, natal dispersal distance ranged from 0.5 to 42 km, with a mean distance of 10.5 km (±6.9 km S.D.). Males and females showed similar dispersal distances (mean dispersal distance for males = 10.3 km (±6.4); mean dispersal for females = 10.6 km (±7.5); Table 2). Similarly, natal dispersal distance was not related to color morph (mean dispersal distance for both gray and brown morph = 10.4 km (±6.8 and ±6.9 respectively) (Table 2, Figure 1b). In our LMM on variation in dispersal distances considering the larger sample of recruits (*n* = 180; see Table 1), we found a significant effect of the interaction between color and winter temperature: in colder winters gray individuals dispersed further away from their native territory than brown ones, whereas in milder winters the brown morph dispersed longer distances than gray ones (SE = 2.37 (±1.14), F_1,166_ = 4.35, *P* = 0.039; Table 2a; Figure 2). Moreover, in this model, we found that age at recruitment did not affect dispersal distance (Table 2a). The model including only one-year-old individuals confirmed a strong morph-specific effect of winter temperature on dispersal distance (SE = 3.59 (±1.36), F_1,70_ = 6.96, *P* = 0.010; Table 2b; Supplementary Figure S2). In this model, we also found a negative relationship between dispersal distances and body mass at fledging where individuals with higher body mass dispersed shorter distances (SE = −1.80 (±0.72), F_1,77_ = 6.52, *P* = 0.015; Table 2b; Supplementary Figure S3). On the other hand, we did not find support for an effect of small mammal abundance on dispersal distance (Table 2a–b).

**Table 2:** Linear mixed model analyzing variation in natal dispersal distances a) in all the individuals recruited in the tawny owl population (*n* = 180) and b) among 1-year-old recruits (*n* = 84). Model includes both environmental variables on annual level (winter temperature anomaly (see Material and methods for variable explanation), mammal prey abundance) as well as individual specific traits (color morph, age at recruitment, sex, and body mass at fledging) as fixed terms. For the class variables color morph, age at recruitment, and sex: “gray”, “1 year-old” and “female” are used as reference level respectively. Year, “brood ID” and “nest box ID” are entered as random factors. Bold font indicates significant fixed terms

a) Dependent Variable	Predictors	Estimate	SE	df	F	P
Dispersal distance (*n* = 180)	Intercept	10.39	0.96	166.92	–	–
	Age at recruitment (2-year-old)	0.73	1.23	170.93	0.33	0.718
	(3-year-old)	0.98	1.32	168.87		
	Color morph (brown)	0.27	1.13	170.60	0.06	0.810
	Body mass at fledging	−0.81	0.62	166.73	1.72	0.191
	Sex (male)	−0.93	1.17	170.41	0.64	0.425
	Mammal prey abundance	−0.25	0.57	165.36	0.19	0.667
	Winter temperature anomaly	−0.85	0.31	170.90	0.90	0.344
	**Winter temperature anomaly:color morph**	**2.37**	**1.14**	**166.31**	**4.35**	**0.039**
Random factors	Year: Variance <.0001					
	Brood ID: Variance <.0001 Nest box ID: Variance = 3.382, SD 1.84					
b) Dependent Variable	Predictors	Estimate	SE	df	F	P
Dispersal distance (*n* = 84)	Intercept	10.90	0.96	75.00	–	–
	Color morph (brown)	0.95	1.53	76.96	0.39	0.536
	**Body mass at fledging**	−**1.80**	**0.72**	**76.61**	**6.25**	**0.015**
	Sex (male)	−2.14	1.40	76.97	2.34	0.130
	Mammal prey abundance	0.31	0.67	76.77	0.22	0.639
	Winter temperature anomaly	−1.68	0.84	75.95	0.02	0.879
	**Winter temperature anomaly:color morph**	**3.59**	**1.36**	**69.81**	**6.96**	**0.010**
Random factors	Year: Variance <.0001					
	Brood ID: Variance <.0001 Nest box ID: Variance = 2.39, SD 1.55					

### Detectable distances versus observed distances

**Figure 3:**
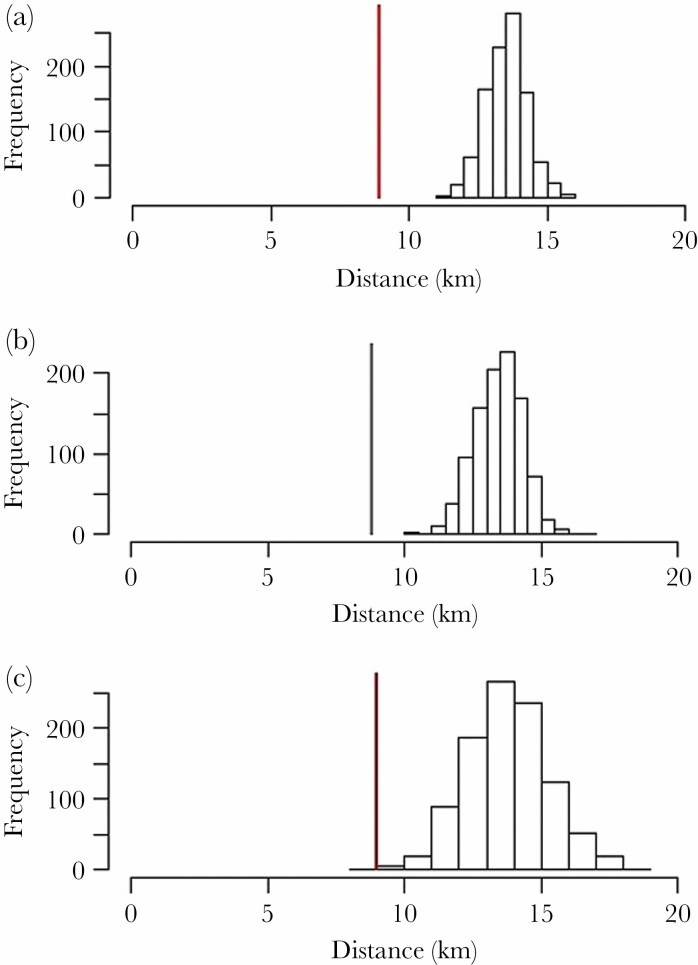
Histograms showing the distribution of observed median distances and the expected (random) median dispersal distances based on 999 draws assuming random natal dispersal in a) all the recruited individuals in the sample (*n* = 192) (observed median distance in red), b) gray morph recruits (*n* = 134) (observed median distance in gray) and c) brown morph recruits (*n* = 56) (observed median distance in brown). The distribution of expected distances is slightly wider in brown compared with gray because of the lower sample size. In each case, observed dispersal distance clearly falls below the expected distances (a–b: *P* < 0.001, c: *P* = 0.002)..

The randomization test of possible detectable distances showed that pairwise distances between nest boxes were most commonly between 10–15 km in our study area (Supplementary Figure S4), indicating that the study area was adequately sized as the observed dispersal clearly fell below the expected distances (*P* < 0.001, see Figure 3a). This pattern was maintained also when we compared separately detectable and observed distances for gray and brown morph (*P* < 0.001 and *P* = 0.002 respectively, see Figure 3b–c).

## DISCUSSION

Our results provide novel insight on drivers of variation in dispersal in tawny owls at their northern range margin, and more specifically how environmental conditions shape the natal dispersal of heritable phenotypes. Contrarily to what was observed in barn owls (van den Brink et al. 2012a), we find that genetically determined color polymorphism *per se* is not directly linked with variation in dispersal distances in tawny owls, but rather, that winter temperature differentially affects the dispersal of the two tawny owl morphs. We expected that tawny owl morphs differ in their dispersal patterns according to winter conditions. Indeed, we found that in colder winters, individuals of the gray morph dispersed longer distances than brown individuals, whereas in milder winters the brown morph undertook the longer movements.

A review of the conditions under which brown or gray tawny owls are suggested to perform the best (Emaresi et al. 2011) highlighted that the brown morph would have a higher fitness under less stressful situations (e.g. warm-wet climate conditions and high food availability), whereas the gray morph would perform better in stressful conditions (e.g. cool-dry years and low-food availability), potentially due to morph-specific intrinsic metabolic differences (Piault et al. 2009). Only about 6% of the fledged tawny owls recruit back into the population in our study area, with a large difference in recruitment between years due to variation in winter climate (Morosinotto et al. 2020). It is possible that the pattern here observed of longer dispersal distances of gray individuals after cold winters are linked to survival selection acting against brown (but not gray) tawny owls that disperse farther. When winters are severe, the gray morph enjoys advantages over the brown morph as it is better camouflaged in snowy winter conditions (Koskenpato et al. 2020), has a better plumage insulation capacity (Koskenpato et al. 2016) and has overall higher survival (Karell et al. 2011b). Therefore, the gray morph may have a higher probability to survive longer dispersal movements under harsh climatic conditions compared with the brown morph. Unfortunately, our data do not allow assessment of dispersal distance of non-survivors which would be required to test this hypothesis.

Another potential mechanism behind our findings is based on morph-specific differences in competitiveness over breeding territories. The tawny owl is known to be highly territorial and philopatric (Hirons 1985, Sunde 2011), but nothing is known about possible differences in the degree of territoriality between morphs. We would expect that brown tawny owls are better competitors for territories because they have a more aggressive personality (Da Silva et al. 2013). After cold and snow-rich winters, more territories become vacant as mortality among territorial owls is high in harsh winters (Francis and Saurola 2002, Karell et al. 2011b). Under these conditions, and if brown individuals are more territorial and more aggressive competitors, brown tawny owls may outcompete gray ones for the closest vacant territories. Increased vacancy of territories after cold winters might therefore allow brown tawny owls to avoid the costs entailed by moving further under unfavorable conditions because the costs would probably be higher for brown than gray ones given their lower tolerance to cold environments (Karell et al. 2011b). In fact, even relatively short distances might have an impact on this site-tenacious species when associated with, for instance, scarce knowledge of surroundings and hunting grounds as well as competition with territorial adults and other floaters that in turn could reinforce the effect of adverse winter conditions. On the other hand, when winters are milder, and hence conditions are more favorable but fewer territories are vacant, brown individuals are able to pay the costs of moving farther away from their natal territory and disperse larger distances. However, we are not able here to discuss how dispersal can affect survival, as we cannot assess if morphs pay a higher cost when they decide to stay closer to their natal territory or when they undertake a longer displacement.

Measurements of natal dispersal in studies of natural populations are commonly defined as the observed distance an identifiable individual has traveled from the natal site to the location of its first reproductive attempt (Clobert et al. 2012). However, the probability of detecting dispersers and the distance they travel is strongly determined by the size of the study area (Koenig et al. 1996, Sutherland et al. 2000). We found that the median distances detectable within the study area were clearly longer than the average dispersal distances traveled by the observed recruits (see Figure 3a–c). Our simulation, therefore, shows that both gray and brown tawny owls dispersed clearly shorter distances than what we could expect from a random draw. Therefore, we do not expect that either morph would have a higher likelihood of dispersing out of the study area, and we, therefore, conclude that our measures of dispersal distance are adequate to test our hypothesis. Finally, we cannot discard that natal dispersal in our population could be affected by attributes of habitat that were not taken into account in this study and that could be sensitive to climate variation and/or have an overwhelming effect (Pärn et al. 2012).

Contrarily to our prediction that heavier individuals should disperse further, we observed a negative significant association between body mass at fledging and natal dispersal distance when considering data on one-year-olds only. This seems to support the hypothesis that heavier individuals are superior competitors in acquiring a territory, whereas lighter individuals are dispersing farther to find vacant territories (Becker and Bradley 2007, Cox and Kesler 2012). The pattern was similar in the larger data set including individuals which recruit at an older age, yet not significant. This may be due to the fact that young tawny owls frequently occupy and defend the territory even though they only start to breed for the first time when two or three years old (Sunde 2008, Karell et al. 2009, Millon et al. 2010, Sasvári and Hegyi 2011). This is supported by the lack of relationship between age at recruitment and dispersal distances. However, we cannot speculate about the possible reasons for this because practically nothing is known about the behavior of juveniles and floaters in this system. We can also not fully rule out that the non-significant effect of body mass in older recruits may stem from a diluted effect of environmental conditions among years.

Our finding that annual variation in prey abundance did not explain dispersal distances may be due to the low survival of juveniles in years of low prey abundance. Most dispersers might simply die of starvation if food is scarce and their hunting skills are not yet fully developed (Hirons 1985, Sunde 2005, Millon et al. 2014). Because our data consists only of surviving juveniles which recruit to the population, we have very limited data from years with very poor prey conditions. Therefore, with our data we can only estimate dispersal of those who survive, and among these individuals there is no significant effect of small mammal prey abundance on natal dispersal distance.

We did not find any sex-related pattern in dispersal distances, although an extensive body of work has provided evidence for sex biased dispersal in many birds (e.g. Pusey 1987, Trochet et al. 2016). In tawny owls, both sexes are highly territorial and defend their territory all year round (Hirons 1985, Saurola 1987, Appleby et al. 1999, Sunde 2011), so the need of finding vacant territories is likely equally important in both sexes.

Regardless of the mechanism behind our findings, this study shows how dispersal can be shaped by the interaction between genetically determined phenotypes and their environment. Such insight about the factors influencing dispersal tactics is important because dispersal strategies can have a great impact on other important life-history traits like reproductive success (e.g. Tannerfeldt and Angerbjörn 1996, Steiner and Gaston 2005, Tarwater and Beissinger 2012, Green and Hatchwell 2018). Furthermore, our results support the general prediction that melanin-based morphs are differently affected by climate variation (Roulin 2014). Because color polymorphism is genetically determined, morph-specific dispersal distances regulated by temperature, as we find here in the tawny owl, imply that the rate of spread of genetic variants might change as climate changes. In synergy with climate-driven selection processes acting on color polymorphism (Karell et al. 2011b, Teplitsky and Charmantier 2019) climate-driven dispersal differences between morphs may impact the distribution of morphs, especially in the range margins (Amar et al. 2014, McLean and Stuart-Fox 2014). More emphasis should be placed on the linkage between environment and phenotype rather than only on the direct effects of climate change on dispersal (McCauley and Mabry 2011). Thus, the study of the nexus between climate change, phenotype, and dispersal is of great importance to predict the long-term consequences of climate change on population distribution patterns.

## Supplementary Material

arab149_suppl_Supplementary_Material

## Data Availability

Analyses reported in this article can be reproduced using the data provided by Passarotto et al. (2021).

## Notes

We thank all members of Kimpari Bird Projects (KBP) and Veikko Tarsa, as well as Katja Koskenpato, Ruslan Gunko, Kati Schenk, and Petri Valo for their help with collecting field data on the tawny owls. This is publication number 21 from KBP. Finally, we thank the editor and three anonymous reviewers for their constructive suggestions that improved the manuscript.
