# Supplementary material for: Cold winters have morph-specific effects on natal dispersal distance in a wild raptor
Source: Behav Ecol. 2021 Dec 30;33(2):419–27. doi: 10.1093/beheco/arab149 (PMC9015216; doi:10.1093/beheco/arab149)

Electronic supplementary material for

**Cold winters have morph-specific effects on natal dispersal distance in a wild raptor**(Includes 4 figures)

**
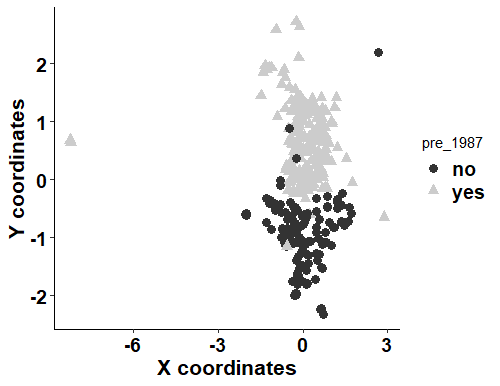
Figure S1.** Scatterplot depicting the distribution of the nest boxes within the two neighboring study areas. Light gray triangles indicate nest boxes present in the area since 1978 while darker dots indicate the nest boxes added from 1987, when the area was enlarged (see main text for study area description). Coordinates are standardized to zero mean.

**Figure S2.**  Dispersal distances travelled by gray and brown one-year-old recruited individuals in relation to winter temperature anomaly (see methods). Lighter gray dots indicate gray individuals (n = 65) while darker gray dots indicate brown individuals (n = 19). The plot presents the marginal effects (± 95 CI) of the interaction in the statistical model (see Table 2b).


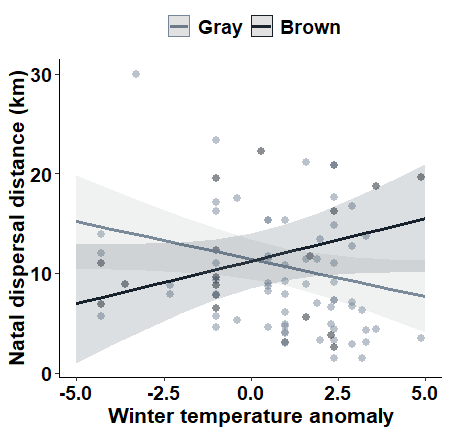


**Figure S3.** Scatterplot depicting the dispersal distances traveled by one-year-old recruited individuals in relation to their body mass at fledging (n = 84). The plot presents the marginal effects (± 95 CI) in the statistical model (see Table 2b in the main text).

**
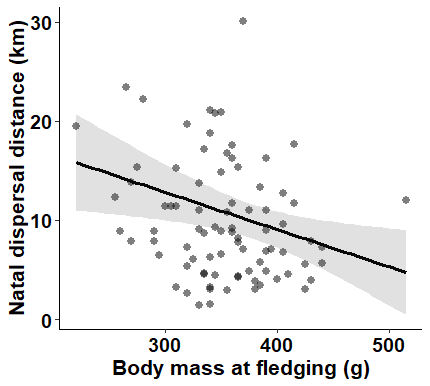
**

**Figure S4.** Distribution of all possible pairwise distances between the nest boxes in the study area. Note that distances 10-15 km are the most common.


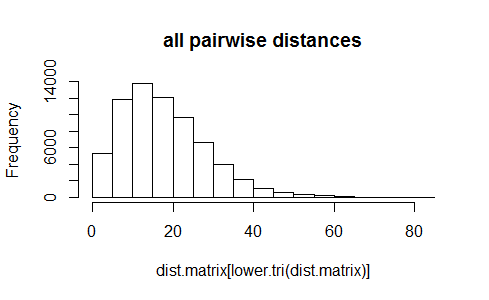

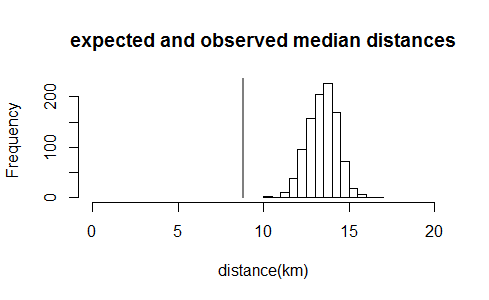

Supplement: arab149_suppl_Supplementary_Material [file arab149_suppl_supplementary_material.docx]
